# SOCIAL CAPITAL AND HOME- AND COMMUNITY-BASED SERVICE UTILIZATION AMONG URBAN OLDER CHINESE ADULTS

**DOI:** 10.1093/geroni/igz038.2372

**Published:** 2019-11-08

**Authors:** Changmin Peng, Jeffery A Burr, Kyungmin Kim, Nan Lu

**Affiliations:** 1 Umass Boston, Boston, Massachusetts, United States; 2 University of Massachusetts Boston, Boston, Massachusetts, United States; 3 Renmin University of China, Beijing, China

## Abstract

Home- and Community-Based Services (HCBS) are increasingly important for older adults who want to maintain their independence and remain in their communities. Although HCBS systems have been developed widely in many western countries and in some countries in Asia, China is just beginning to grapple with its rapidly aging population by offering HCBS in a limited fashion. The purpose of this study was to investigate the relationship between structural (e.g., citizenship activities, volunteering) and cognitive (e.g., social trust, a sense of belonging) social capital and HCBS utilization among older Chinese adults. The study also examined the mediating effect of structural social capital for the the relationship between cognitive social capital and HCBS utilization. We frame the study within the Andersen behavioral model of health services utilization and argue that within this framework social capital is an enabling factor. We analyzed survey data from 456 community-dwelling older adults living in the Gusu district of the city of Suzhou, China in 2015. Structural equation modeling was used to test the hypothesized relationships. The results showed that both cognitive and structural social capital were significantly associated with HCBS utilization. Structural social capital also served as a mediator between cognitive social capital and HCBS utilization, even after controlling for sociodemographic characteristics and other relevant covariates. The findings supported the utility of employing Andersen’s behavioral model and social capital theory for better understanding older Chinese adults’ utilization of HCBS. Interventions for increasing social capital may be useful for improving HCBS utilization in Chinese urban communities.

